# Characteristics of isoniazid-induced psychosis: a systematic review of case reports and case series

**DOI:** 10.1007/s00228-024-03738-x

**Published:** 2024-08-13

**Authors:** Keerthanaa B, Rashmi Appaji, Levin Thomas, Tejaswini Baral, Skanda N, Chaithra, Sonal Sekhar M, Kavitha Saravu, Krishna Undela, Mahadev Rao

**Affiliations:** 1 Department of Pharmacy Practice, Manipal College of Pharmaceutical Sciences, Manipal Academy of Higher Education, Manipal, 576104 Udupi, Karnataka India; 2 Department of Psychiatry, Father Muller Medical College Hospital, Father Muller Road, Kankanady, 575002 Mangalore, Karnataka India; 3 Department of Infectious Diseases, Kasturba Medical College, Manipal, Manipal Academy of Higher Education, Manipal, 576104 Udupi, Karnataka India; 4 Department of Pharmacy Practice, National Institute of Pharmaceutical Education and Research (NIPER), Guwahati, Changsari, Kamrup (R), Assam 781101 India

**Keywords:** Adverse drug reaction, Isoniazid, Psychosis, Pyridoxine, Tuberculosis

## Abstract

**Purpose:**

Isoniazid, a first-line antitubercular drug, is associated with nervous system adverse drug reactions such as seizures, peripheral neuropathy, and psychosis. This systematic review of case reports and case series aimed to characterize the demographic, social, and clinical factors associated with isoniazid-induced psychosis in patients with active tuberculosis (TB) and those who received isoniazid for latent TB infection (LTBI).

**Methods:**

We comprehensively searched the Embase, PubMed, and Scopus databases to identify relevant studies published between the date of inception of the database and June 2024.

**Results:**

A total of 28 studies, including 21 case reports and 7 case series involved 37 patients who developed isoniazid-induced psychosis. A higher frequency of isoniazid-induced psychosis was observed during the first 2 months of treatment, with a relatively early onset observed among patients aged 18 years or less. Delusions and/or hallucinations are the common symptoms of isoniazid-induced psychosis. Psychomotor disturbances, disorganized speech or formal thought disorder, disorganized or abnormal behaviour, and neuropsychiatric symptoms (sleep disturbances, hostility or aggression, confusion, affective symptoms, anxiety symptoms, and cognitive difficulties) were the other symptoms observed in the included studies. More than 80% of cases rechallenged with isoniazid resulted in the recurrence of psychotic symptoms.

**Conclusion:**

Patients with TB and LTBI should be assessed for psychotic and neuropsychiatric symptoms during isoniazid therapy, mainly in the first 2 months. Further research is required to understand the impact of underlying risk factors, such as genetic predisposition and isoniazid pharmacokinetics, as well as the clinical utility and dosage recommendations of pyridoxine for managing isoniazid-induced psychosis.

**Supplementary Information:**

The online version contains supplementary material available at 10.1007/s00228-024-03738-x.

## Introduction

Isoniazid has been one of the first-line antitubercular drugs used to treat both active tuberculosis (TB) and latent TB infection (LTBI) for several years [1]. Isoniazid is associated with numerous types of adverse drug reactions (ADRs) affecting the central and peripheral nervous system, such as insomnia, headache, muscle twitching, optic neuropathy, peripheral neurotoxicity, psychosis, and restlessness [2]. Isoniazid may cause psychiatric ADRs that include symptoms such as delusions, hallucination, abnormal behaviour, disorganized thoughts, and euphoria [3, 4]. Several case studies have found an association between the development of psychosis and isoniazid use in the treatment and prophylaxis of TB [5, 6]. Several mechanisms have been hypothesized for isoniazid-induced psychosis. One mechanism involves the marginal inhibition of monoamine oxidase (MAO) by isoniazid, which results in elevated levels of monoamines [7, 8]. Another mechanism is the lack of vitamin B6/pyridoxine, a cofactor necessary to produce numerous neurotransmitters, including gamma-aminobutyric acid (GABA) [9]. When isoniazid is metabolized, it produces hydrazine and its metabolites (reactive nitrogen species), which bind with pyridoxal phosphate, an active form of pyridoxine. This binding inhibits the enzymes dependent on pyridoxal phosphate, including transaminases and those involved in amino acid metabolism, leading to a functional pyridoxine deficiency [10]. In addition, isoniazid is linked to increased oxidative stress caused by increased production of reactive oxygen species (ROS), which lowers the density of N-methyl-D-aspartate (NMDA) receptors in the hippocampus. ROS are hypothesized to be produced more often, and subsequently, glutathione levels are depleted [11].

The incidence of isoniazid-induced psychosis has not yet been established. The risk factors for the occurrence of psychosis induced by isoniazid include older age, malnourishment, alcohol consumption, diabetes mellites, uraemia, present, past, and family history of psychiatric illness, hepatocellular dysfunction, and neurological disorder [12–14]. Additionally, the isoniazid dose (> 5 mg/kg) and *N* acetyltransferases (*NAT2*) slow acetylators status might contribute to the risk of developing psychosis secondary to isoniazid intake [13]. To our knowledge, no epidemiological studies focused on psychosis due to isoniazid for TB treatment and/or LTBI, and most of the available literature includes case reports and case series.

Despite its clinical importance, there is a limited understanding regarding the demographic, social, and clinical determinants of isoniazid-induced psychosis in patients with TB and LTBI. Our systematic review focused on the in-depth clinical profiling of patients who developed isoniazid-induced psychosis. In this systematic review, we assessed the occurrence of isoniazid-induced psychosis based on patient demographics, social factors, and clinical determinants. We investigated whether there were specific periods during which psychosis was more likely to occur in TB and LTBI patients. This review also provides an overview of the pharmacological management of isoniazid-induced psychosis.

## Methods

### Research question

We conducted our systematic review to address the main research question: What demographic, social, and clinical variables are associated with isoniazid-induced psychosis in patients with active TB or those who received isoniazid for LTBI?

### PICO framework

The population-intervention-comparator-outcomes-study design (PICO) framework was used to identify eligible cases [15]. The criteria used were as follows:

- Population: Active TB/LTBI patients, regardless of age or demographics.
- Intervention: Individuals received isoniazid for active TB treatment/LTBI.
- Comparator: Comparator was not applicable.
- Outcome: The development of psychosis following the initiation of isoniazid therapy as prophylactic or antitubercular treatment.

### Eligibility criteria

- Inclusion criteria: Case studies of isoniazid-induced psychosis was included based on the diagnostic criteria for substance/medication-induced psychosis defined by the Diagnostic and Statistical Manual of Mental Disorders, Fifth Edition (DSM-5) classification [16], published in the format of full text, correspondence, and letters to the editor focused entirely on the case report.
- Exclusion criteria: Review articles, conference abstract, articles not published in the English language, cases with drug-induced psychosis other than isoniazid, patients with a current or previous history of psychiatric illness, and patients with no symptoms of either hallucination or delusion.

### Information source and search strategy

**Fig. 1:**
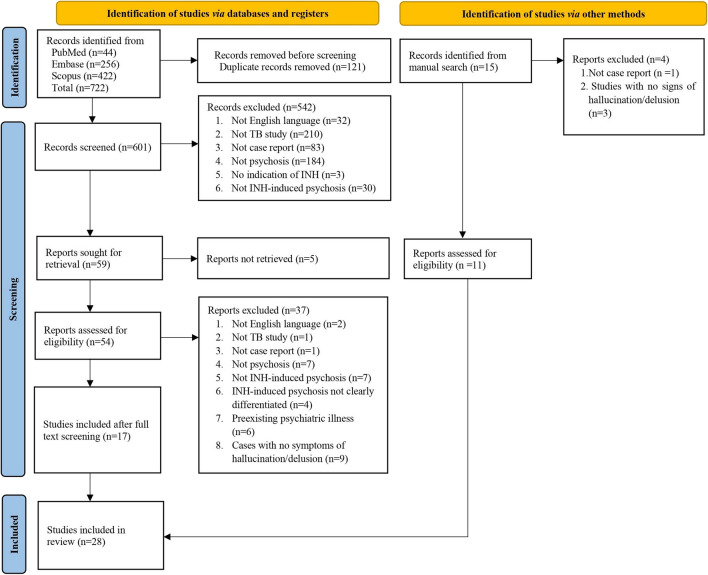
PRISMA 2020 flow diagram for study selection. Abbreviations: INH, isoniazid; PRISMA, Preferred Reporting Items for Systematic Reviews and Meta-Analyses; TB, tuberculosis

The current study was performed according to the Preferred Reporting Items for Systematic Reviews and Meta-Analyses 2020 (PRISMA) guidelines [17] and the PRISMA checklist in Supplemental Table 1. A systematic search approach was used to identify relevant studies. This included searching electronic databases such as PubMed, Embase, and Scopus and manually reviewing the reference lists of pertinent papers. The search was performed from the date of inception of each database until June 2024. The search keywords used in this systematic review are listed in Supplemental Table 2. Figure 1 illustrates the PRISMA flowchart generated using Microsoft Word.

Two independent reviewers, K.B. and S.N., performed article screening and review. Following an initial search and elimination of duplicates, two independent reviewers (K.B. and S.N.) conducted an extensive eligibility evaluation on the titles and abstracts of the remaining articles. The selected articles were subjected to a systematic full-text screening process, and only those that matched the predetermined inclusion criteria were included in the systematic review. When there were disagreements between the two reviewers regarding the acceptability of an article for inclusion, the other reviewers (L.T. and R.A.) were consulted for arbitration. All excluded reports and the reasons for their exclusion were well-documented, ensuring a rigorous and open selection process.

### Selection process and data extraction

Three authors (K.B., L.T., and S.N.) independently extracted the data from each study using a Microsoft Excel spreadsheet. If there was a disagreement among the authors regarding the extracted data, a co-author (R.A. or M.R.) was consulted to arrive at a final decision.

**Fig. 2:**
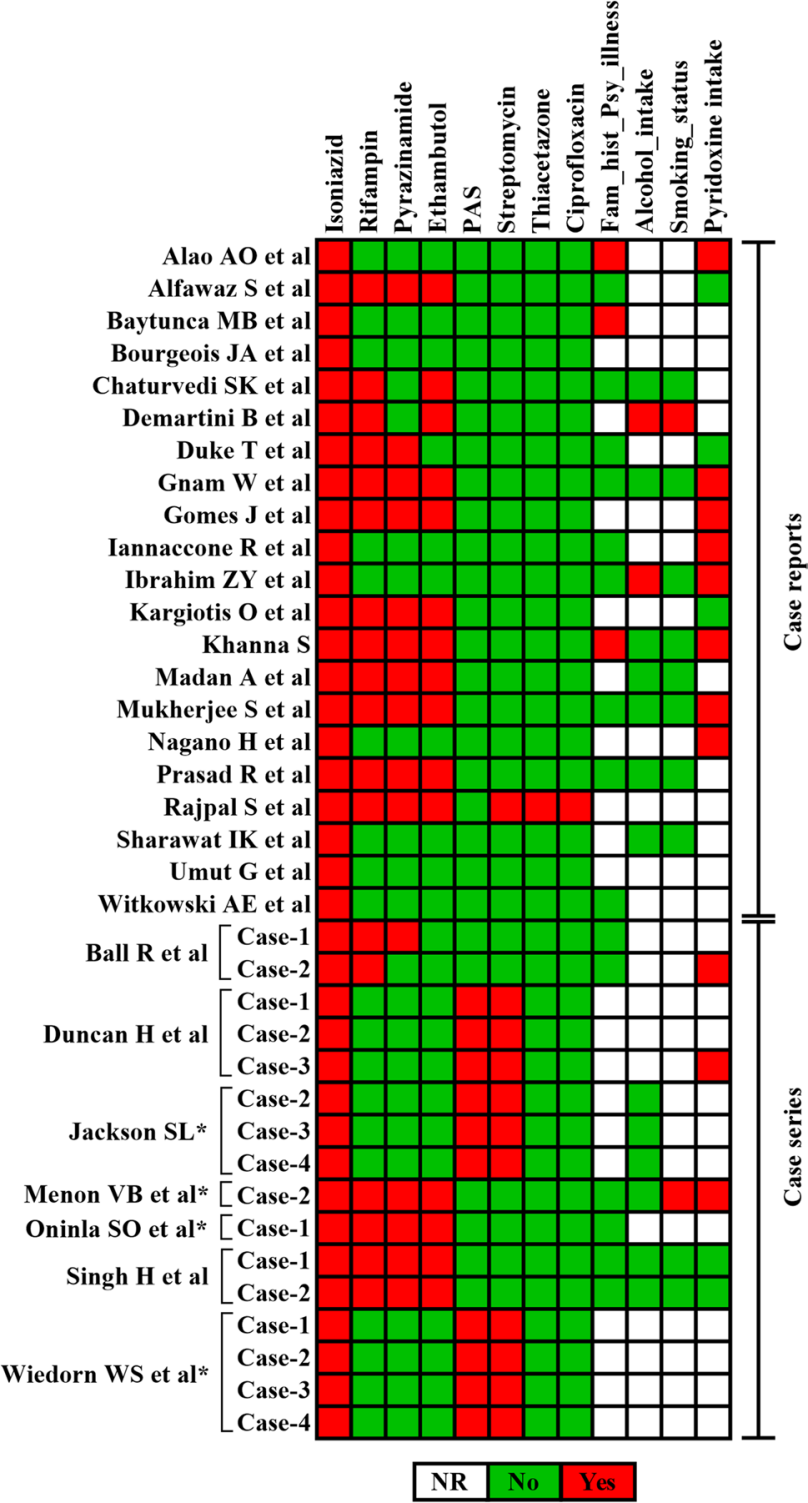
Medication, family and social history profile of patients who developed psychosis after the administration of isoniazid. *Cases that were not isoniazid induced psychosis has been excluded from the case series. Pyridoxine administered before the occurrence of the psychosis. Abbreviations: Fam_hist_Psy_illness, family history of psychiatric illness; NR, not reported; PAS, para-aminosalicylic acid

**Fig. 3:**
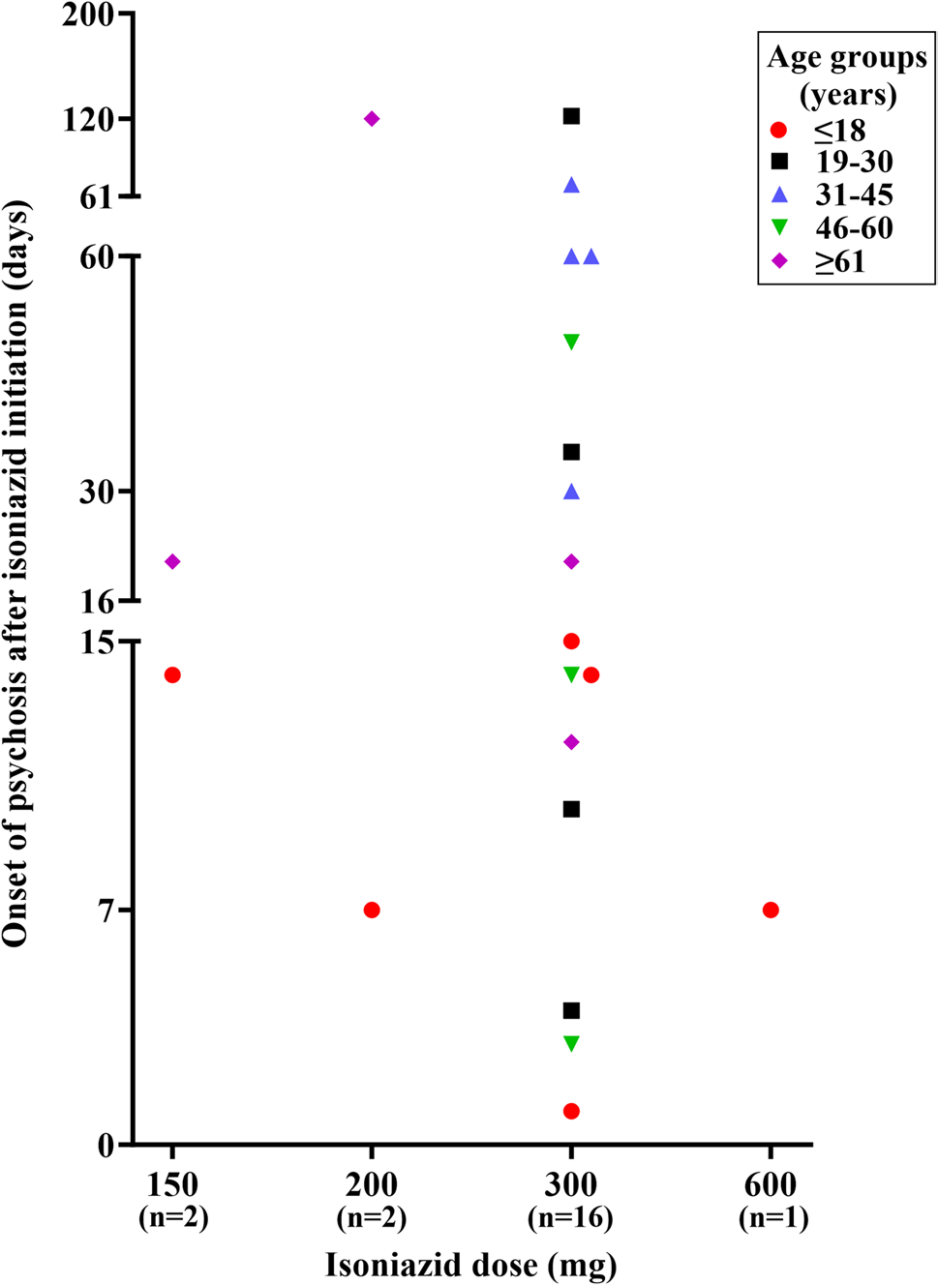
Onset of psychosis symptoms with a dose of isoniazid. Footnote: Out of the 37 observed cases, only 21 were included in this study, while the remaining 16 were excluded due to incomplete documentation of dose or onset of psychosis. Abbreviation: mg, milligram

The data extraction involved demographic, social, and clinical variables from the included studies. The variables were as follows: age, sex, body mass index (BMI), type of TB, treatment regimen, comorbidities/previous medical history, alcohol and smoking history, family history of psychosis/psychiatric illness, onset of psychosis since antitubercular treatment initiation, reported psychotic signs and symptoms, rechallenge with isoniazid and its response, pyridoxine dose administered during or after antitubercular treatment, psychotropics given after psychotic episode, withhold/discontinuation of isoniazid, and resolution of psychotic symptoms following therapeutic action. All extracted data were entered into Microsoft Excel for further analysis. We grouped the included studies as follows: cases prescribed only pyridoxine, cases prescribed only psychotropics, cases prescribed pyridoxine and psychotropics, and cases not prescribed or not mentioned regarding pyridoxine and psychotropic administration. GraphPad Prism Version 10.1.2 (GraphPad Software, USA) was used to plot the assessment of the medication, family and social history profile of patients who developed psychosis after the administration of isoniazid (Fig. 2), the relationship between isoniazid dose and age group with the onset of psychosis (Fig. 3), the symptoms experienced by patients who developed isoniazid-induced psychosis (Supplemental Fig. 1), and the risk of bias of studies (Supplemental Fig. 2).

### Quality assessment

The methodological assessment of study quality was performed using a standardized tool adapted from Murad et al. [18]. This scale assesses selection, ascertainment, causation, and reporting domains. Although there were eight questions, question seven was omitted because it was irrelevant to our review. Therefore, the maximum possible score is seven. Each of the seven items in the questionnaire received a binary (yes/no) response. If the criteria were satisfied, a score of one was given; otherwise, a score of zero was given. If the study obtained a score of five to seven, three to four, and under three, we considered the chance of bias to be low, moderate, or high risk of bias, respectively (Supplemental Fig. 2). Four independent reviewers (K.B., L.T., T.B., and S.N.) evaluated the quality of the studies. In case of any discrepancies concerning the overall rating of a paper, R.A. acted as an arbitrator.

## Results

### Study selection

A comprehensive search of the electronic databases yielded 722 records. After removing 121 duplicates, 601 records were used for title and abstract screening. We sought 59 studies for retrieval, of which five could not be retrieved because of the unavailability of the full text. Finally, 54 studies were deemed relevant and subjected to a full-text review. After thoroughly evaluating full-text articles consisting of case reports, case series, and cases in the form of letters to the editor or short communications, 17 studies met our inclusion criteria. In addition, 11 more studies were identified by manually searching the articles from pertinent literature references. A total of 28 publications were included in our review [5, 6, 12, 14, 19–42], which consisted of 37 cases from 13 case reports [5, 6, 12, 14, 19, 20, 24, 27, 30, 31, 34, 36, 39], seven case series (16 cases) [21, 25, 32, 35, 37, 38, 40], seven letters to editors [22, 23, 26, 28, 29, 41, 42], and one short communication [33]. The data extracted from the selected studies are presented in Table 1.

**Table 1:** Characteristics of included case reports and case studies

Sl.no	First author; year; country (race/ethnicity/immigrant) [reference]	Age; sex	Study design	BMI (kg/m^2^)	TB/LTBI	Type of TB	Comorbidities/previous medical history	Onset of psychosis diagnosis since ATT initiation	Rechallenge was done	Appearance of psychotic and neuropsychiatric symptoms after the rechallenge of isoniazid	Pyridoxine dose administered during ATT	Pyridoxine dose was administered after psychosis	Psychotropics given after psychotic episode
Cases prescribed only pyridoxine													
1	Duncan H et al.; 1962; UK-Case 1 (Nigerian) [25]	35; F	Case series	NR	TB	EPTB	NR	Fourth week	Yes	Yes	NR	500 mg BD	NR
2	Duncan H et al.; 1962; UK-Case 3 [25]	61; F	Case series	NR	TB	PTB (bilateral apical TB)	Diabetes	3 weeks	No	No	40 mg/day	80 mg/day	NR
3	Iannaccone R et al.; 2002; USA [27]	14; F	Case report	NR	LTBI	–	Generalized tonic–clonic seizures	1 day	No	No	50 mg/day	NR	NR
4	Kargiotis O et al.; 2015; Greece [29]	75; M	Letter to Editor	NR	TB	PTB	Arterial hypertension	3 weeks	No	No	No	50 mg/day	No
Cases prescribed only psychotropics													
5	Ball R et al.; 1989; UK-Case 1 (Hong Kong immigrant) [21]	53; M	Case series	NR	TB	PTB	Insulin-dependent diabetes	2 weeks	No	No	NR	NR	Chlorpromazine
6	Baytunca MB et al.; 2015; Turkey [22]	11; F	Letter to Editor	NR	LTBI	–	No	2 weeks	No	No	NR	NR	Risperidone Fluoxetine Aripiprazole
7	Demartini B et al.; 2013; Sri Lanka [23]	56; M	Letter to Editor	NR	TB	EPTB	Hepatic encephalopathy	NR	No	No	NR	NR	Delorazepam Haloperidol
8	Prasad R et al.; 2008; India [14]	28; M	Case report	NR	TB	PTB	No	4 days	Yes	Yes	NR	NR	Diazepam
9	Rajpal S et al.; 2000; India [36]	18; M	Case report	NR	TB	PTB	NR	8 months	No	No	NR	NR	Drug name not mentioned
10	Singh H et al.; 2017; India-Case 2 [37]	51; F	Case series	NR	TB	Miliary TB	AKI, ATT-hepatitis	1 week	No	No	No	No	Lorazepam, Risperidone
11	Umut G et al.; 2016; Turkey [39]	28; M	Case report	NR	LTBI	–	Ankylosing spondylitis	10 days	No	No	NR	NR	Quetiapine Haloperidol
Cases prescribed pyridoxine and psychotropics													
12	Alao O et al.; 1998; USA [19]	31; F	Case report	NR	LTBI	–	No	8 weeks	No	No	50 mg/day	NR	Olanzapine
13	Alfawaz S et al.; 2020; Saudi Arabia [20]	12; F	Case report	11.98	TB	PTB	No	2 weeks	No	No	Not prescribed	25 mg/day	Risperidone
14	Ball R et al.; 1989; UK-Case 2 (Filipino immigrant) [21]	43; F	Case series	NR	TB	EPTB	NR	2 months	No	No	Yes-dose unknown	Yes-dose unknown	Thioridazine
15	Chaturvedi SK; 1988; India [42]	18; M	Letter to Editor	NR	TB	PTB	NR	15 days	Yes	Yes	NR	Yes-dose unknown	Haloperidol
16	Duke T et al.; 1999; Papua New Guinea [24]	6; M	Case report	NR	TB	EPTB	No	2 weeks	Yes	Yes	No	5 mg daily-first visit 10 mg after rechallenging	Chlorpromazine
17	Gnam W et al.; 1993; China [26]	64; F	Letter to Editor	NR	TB	PTB	Optic atrophy	3 months	No	No	25 mg/day	25 mg/day	Loxapine Perphenazine
18	Gomes J et al.; 2019; Portugal (African descent) [12]	21; F	Case report	NR	TB	EPTB	No	4 days	No	No	200 mg/day	NR	Olanzapine
19	Ibrahim ZY et al.; 1994; USA [28]	64; M	Letter to Editor	NR	LTBI	–	Seizure disorder Anaemia Glaucoma Bell’s palsy	12 days	No	No	50 mg/day	NR	Haloperidol Lorazepam
20	Khanna S et al.; 2022; India [30]	31; M	Case report	NR	TB	PTB	NR	4 weeks	No	No	20 mg/day	NR	Olanzapine Lorazepam
21	Mukherjee S et al.; 2023; India [33]	47; F	Short communication	NR	TB	PTB	No	3 days	No	No	20 mg/day	No	Olanzapine
22	Madan A et al.; 1989; India [31]	20; M	Case report	NR	TB	PTB	Anaemia	5 weeks	Yes	Yes	NR	100 mg	Diazepam
23	Menon VB et al.; 2017; India-Case 2 [32]	55; M	Case series	18.08	TB	PTB	No	7 weeks	Yes	Yes	20 mg/day	40 mg/day	Lorazepam Haloperidol Risperidone Quetiapine Clonazepam
24	Nagano H et al.; 2019; Japan [34]	80; F	Case report	NR	LTBI	–	Rheumatoid Arthritis Organizing pneumonia Vasospastic angina	4 months	No	No	30 mg	NR	Quetiapine
25	Oninla S et al.; 2016; Nigeria-Case 1 [35]	14; M	Case series	NR	TB	PTB	Undernutrition Anaemia Congestive cardiac failure	9 days	Yes	No	NR	100 mg/day	Haloperidol
26	Singh H et al.; 2017; India-Case 1 [37]	18; F	Case series	NR	TB	PTB	No	1 week	No	No	No	Yes-dose unknown	Risperidone, Lorazepam
27	Witkowski AE et al.; 2007; Philippines [41]	61; F	Letter to Editor	NR	LTBI	–	Abdominal pain Received appendectomy and small bowel resection	3 weeks	No	No	NR	200 mg	Risperidone
Cases not prescribed or not mentioned regarding pyridoxine and psychotropics administration													
28	Bourgeois JA et al.; 1996; USA (Filipino) [6]	24; M	Case report	NR	LTBI	–	NR	4 months	No	No	NR	NR	No
29	Duncan H et al.;1962; UK-Case 2 (Jamaican) [25]	40; M	Case series	NR	TB	EPTB	NR	Not clearly defined	No	No	NR	NR	NR
30	Jackson SL; 1954; Canada-Case 2 [38]	39; M	Case series	NR	TB	PTB	No	10 weeks	No	No	NR	NR	No
31	Jackson SL; 1955; Canada—Case 3 [38]	37; M	Case series	NR	TB	PTB	No	Not clearly defined	No	No	NR	NR	NR
32	Jackson SL; 1955; Canada-Case 4 [38]	17; M	Case series	NR	TB	NR	No	1 weeks	Yes	Yes	No	No	No
33	Sharawat IK et al.; 2021; India [5]	3; F	Case report	NR	LTBI	–	No	2 weeks	No	No	NR	NR	No
34	Wiedorn WS et al.; 1954; USA-Case 1 (African descent) [40]	27; F	Case series	NR	TB	PTB	NR	8 months	No	No	NR	NR	No
35	Wiedorn WS et al.; 1954; USA-Case 2 (African descent) [40]	46; M	Case series	NR	TB	PTB	Peripheral neuropathy Malnutrition	2 weeks	No	No	NR	NR	No
36	Wiedorn WS et al.; 1954; USA-Case 3 (African descent) [40]	31; F	Case series	NR	TB	PTB	NR	4 months	No	No	NR	NR	No
37	Wiedorn WS et al.; 1954; USA-Case 4 (African descent) [40]	26; F	Case series	NR	TB	Disseminated TB	NR	Not clearly defined	No	No	NR	NR	No

Abbreviations: *ADR*, adverse drug reaction; *AKI*, acute kidney injury; *ATT*, antitubercular treatment; *BD*, bis in die; *BMI*, body mass index; *CKD*, chronic kidney disease; *ESRD*, end-stage renal disease; *EPTB*, extrapulmonary tuberculosis; *F*, female; *HIV*, human immunodeficiency viruses; *INH*, isoniazid; *LTBI*, latent tuberculosis infection; *M*, male; *Mg*, milligram; *NR*, not reported; *PTB*, pulmonary tuberculosis; *TB*, tuberculosis

### Characteristics of included studies

Our comprehensive search identified 37 cases in 14 countries. Out of the 37 cases, 10 reported the race/ethnicity/immigrant status of the patients [6, 12, 21, 25, 40] (Table 1). The countries from which case studies were reported include India (*n* = 10) [5, 14, 30–33, 36, 37, 42], the USA (*n* = 8; 4 African descent, 1 Filipino, and 3 unknown) [6, 19, 27, 28, 40], the UK (*n* = 5; 1 Hong Konger, 1 Filipino, 1 Jamaican, 1 African descent, and 1 unknown) [21, 25], Canada (*n* = 3) [38], Turkey (*n* = 2) [22, 39], China (*n* = 1) [26], Nigeria (*n* = 1) [35], Greece (*n* = 1) [29], Japan (*n* = 1) [34], Papua New Guinea (*n* = 1) [24], the Philippines (*n* = 1) [41], Portugal (*n* = 1; 1 African descent) [12], Saudi Arabia (*n* = 1) [20], and Sri Lanka (*n* = 1) [23].

The median age of the patients were 31 (IQR 34) years (age range from 3 to 80 years). The frequencies of male and female patients with isoniazid-induced psychosis across the cases were 19 (51.3%) and 18 (48.6%), respectively. Of the 37 cases, 19 were reported to have pulmonary TB (PTB) [14, 20, 21, 25, 26, 29–33, 35–38, 40, 42], six were extra-pulmonary TB (EPTB) [12, 21, 23–25], one had disseminated TB [40], and one had miliary TB [37]. Among the reported cases of EPTB, there were cases of pleural effusion TB (*n* = 2) [12, 23], TB meningitis (*n* = 2) [24, 25], spinal TB (*n* = 1) [21], pericarditis TB (*n* = 1) [25]. Furthermore, nine additional cases were where isoniazid was administered for LTBI [5, 6, 19, 22, 27, 28, 34, 39, 41]. However, one case did not report the type of TB [38]. Patients diagnosed with TB also had comorbidities, such as anaemia (*n* = 3) [28, 31, 35] and diabetes mellitus (*n* = 2) [21, 25]. There was one case with a documented history of smoking [32], one with alcohol consumption [28], and one with both smoking and alcohol consumption [23], respectively (Fig. 2). There were three cases in where the patient had a family history of psychosis [19, 22, 30] (Fig. 2). All 37 patients received isoniazid treatment. The combination of antitubercular drugs administered to patients in conjunction with isoniazid is shown in Fig. 2.

Psychosis was diagnosed at a median of 15 days (IQR 46) after the initiation of isoniazid (*n* = 37). The dose of isoniazid reported in these studies was between 150 and 600 mg daily. Among the 37 patients included in the study, the most frequently reported dose of isoniazid was a 300 mg daily (*n* = 17). Assessment of isoniazid daily dose and age groups with the onset time of psychosis (*n* = 21) revealed that all patients ≤ 18 years developed isoniazid-induced psychosis within 15 days of isoniazid initiation, as shown in Fig. 3.

Approximately 75.8% (*n* = 25) of patients developed psychosis within the first 60 days of isoniazid therapy. From our findings, we found that the use of pyridoxine during antitubercular treatment has been documented in 11 cases (Fig. 2), whereas 13 cases have reported pyridoxine supplementation following the onset of psychosis. The total daily dose of pyridoxine ranged from 5 to 1000 mg. Delusions (*n* = 27), hallucinations (*n* = 26), psychomotor disturbances (*n* = 25), disorganized speech (or) formal thought disorder (*n* = 19), disorganized (or) abnormal behaviour (*n* = 15), sleep disturbances (*n* = 13), hostility (or) aggression (*n* = 10), confusion (*n* = 11), affective symptoms (*n* = 10), anxiety symptoms (*n* = 9), and cognitive difficulties (*n* = 7) were observed in patients with isoniazid-induced psychosis (Supplemental Fig. 1).

From the selected 37 cases, we observed that after the onset of isoniazid-induced psychosis, the isoniazid was dechallenged in 32 cases [5, 6, 12, 14, 19–21, 23–25, 28–42]. Of these 32 cases, in 19 cases, patients who received psychotropics and had complete resolution of psychotic and neuropsychiatric symptoms [12, 14, 19–21, 23, 24, 28, 30–37, 39, 41, 42], whereas in 13 patients who did not receive psychotropic intervention [5, 6, 25, 29, 38, 40], nine had complete resolution of the psychotic and neuropsy**c**hiatric symptoms [5, 6, 25, 29, 38, 40], two had a partial resolution [40], and two had no improvement in their psychotic/neuropsychiatric symptoms [38]. In one of the cases with partial resolution of symptoms, cognitive disturbance in the form of disorientation persisted, although psychotic symptoms such as auditory and visual hallucinations resolved after 3 months. In this case, the patient had a medical history of peripheral neuropathy and malnutrition. There were no reports of social history (alcohol consumption and smoking habits) or a family history of psychiatric illness. In another case, the patient experienced disturbance even after 8 weeks of discontinuing isoniazid, and there were no reports of any comorbid conditions, social history, or family history of mental illness. Neither patients were rechallenged with isoniazid or prescribed pyridoxine (both prophylactically and therapeutically) and psychotropics [40]. In our review, out of 37 cases, isoniazid was not dechallenged in five cases [21, 22, 26, 27, 37], of which four received psychotropics [21, 22, 26, 37], while one did not receive psychotropic intervention [27] and had complete resolution of the symptoms. Patients were treated with haloperidol (*n* = 6), lorazepam (*n* = 5), risperidone (*n* = 6), olanzapine (*n* = 4), quetiapine (*n* = 3), and clonazepam (*n* = 1), either as monotherapy or in combination therapy for managing psychiatric symptoms (Table 1). Out of 37 cases, eight were rechallenged with isoniazid, of which seven (Table 1) experienced recurrence of psychotic/neuropsychiatric symptoms (Table 1). We found that only five cases were evaluated, which included an assessment of the causality and severity of ADR [30, 32, 33, 37]. The ADR causality assessment tools employed were the World Health Organization (WHO) Uppsala Monitoring Centre (UMC) scale in four cases [32, 33, 37], and the Naranjo scale in four cases [30, 32, 37]. The Hartwig and Siegel severity scale was used in two cases [32, 33]. The grades of the aforementioned scales are listed in Table 2.

**Table 2:** Reported adverse drug reaction causality, and severity assessment grades for isoniazid-induced psychosis from included cases

S.N	Studies		WHO-UMC scale	Naranjo scale	Hartwig ADR severity scale
1	Khanna S et al. [30]		NR	Possible	NR
2	Menon VB et al. [32]	Case 2	Definite	Definite	Moderate
3	Mukherjee S et al. [33]		Probable	NR	Moderate
4	Singh H et al. [37]	Case 1	Possible	Possible	NR
5		Case 2	Possible	Possible	NR

*ADR* adverse drug reaction, *NR* not reported, *WHO-UMC* World Health Organization-Uppsala Monitoring Centre

### Quality of assessment

According to the risk of bias score and classification rules, 25 cases received a score between five and seven (low risk of bias) [5, 12, 14, 19–32, 34–36, 39, 41, 42], six cases received a score between three and four (moderate risk of bias) [6, 25, 33, 37, 38], and six cases received a score of less than three (high risk of bias) [38, 40]. A detailed quality assessment of each case is presented in Supplemental Fig. 2.

## Discussion

Psychosis is a rare but serious adverse event associated with isoniazid therapy [10]. Our systematic review included 37 patients with psychosis after the isoniazid intake. We found that most cases of isoniazid-induced psychosis were reported in India (*n* = 10). India is among the top 30 countries with a high TB burden as per the Global Tuberculosis Report 2022 [43]. We identified several variables such as a family history of mental illness, alcohol and smoking history, comorbidities, and the lack of pyridoxine supplementation among patients with isoniazid-induced psychosis. We excluded the studies reporting current or previous history of psychiatric illness. However, the evidence for these associations between ATT-induced psychosis remains insufficient despite some studies being in line with previous research [7, 44–47]. A recent systematic review and meta-analysis of 53 studies reported a high pooled prevalence (48%) of malnutrition (51 studies used a low BMI to define malnutrition) among PTB patients [48]. Nutritional deficiencies and complications that occur during malnutrition, such as sleep deprivation and metabolic derangements, are known medical causes of psychosis; nutritional rehabilitation and weight restoration may be key interventional strategies for the resolution of psychosis [49]. Lower BMI (< 18.5 kg/m^2^) was reported for the two cases that have BMI data in our systematic review [20, 32]. There is a high prevalence of TB in patients with human immunodeficiency virus (HIV) co-infection [50]. A meta-analysis reported a pooled prevalence of 23% of the first episodes of psychosis in people living with HIV (PLWHIV) on the African continent [51]. Additionally, the occurrence of psychosis has been reported in the treatment with antiretroviral agents [52, 53]. Co-administration of isoniazid preventive therapy and antiretroviral therapy (ART) was reported to be associated with a higher frequency of psychosis [54]. Further studies assessing the possibility of a synergistic effect of isoniazid and antiretroviral agents in causing psychosis among TB patients co-infected with HIV are required. Hence, factors such as lower BMI/malnutrition, HIV co-infection, and co-administration of antiretroviral agents with isoniazid should be considered as potential confounding variables and/or risk factors in future studies.

The recommended daily dose of isoniazid in adults is 5 mg/kg [55]. Our systematic review identified a daily dose of 300 mg of isoniazid as the most frequently prescribed dose among cases who developed isoniazid-induced psychosis [6, 12, 19, 21, 22, 25, 27–33, 38, 39, 42]. *NAT2* genotype is an important covariate influencing the plasma concentrations of isoniazid. *NAT2* slow acetylators achieve higher concentrations of isoniazid than intermediate and fast acetylators [56]. Only one case in our systematic review reported *NAT2* phenotype status as a *NAT2* fast acetylator [34]. *NAT2* single nucleotide polymorphisms (SNPs) and haplotypes were not assessed (or) reported in any of the 37 cases. *NAT2* slow acetylators have been implicated as a risk factor for the development of isoniazid-induced ADRs, such as drug-induced liver injury (DILI), particularly during the first month of isoniazid therapy initiation [57, 58]. Future studies should identify and evaluate whether patients with *NAT2* genotype/SNPs have an increased risk of developing psychosis due to higher plasma concentrations of isoniazid. Most patients developed isoniazid-induced psychosis within 2 months of isoniazid initiation; although in some cases, it appears even after eight months of therapy. Closer monitoring of isoniazid-induced psychotic symptoms in paediatric patients may be required during the first 2 weeks of treatment. Numerous studies have consistently shown a significant association between the utilization of antibiotics such as cephalosporins and fluoroquinolones and the occurrence of psychosis within a remarkably short period of 2 weeks following the initiation of treatment [44, 59–64]. Considering the highly heterogeneous and pleomorphic presentation of isoniazid psychosis, we operationalized the definition based on broad criteria of psychotic disorders as per the International Classification of Diseases (ICD)-10 [65] and DSM-5 [16]. From our review findings, psychotic symptoms (psychomotor disturbance, disorganized speech (or) formal thought disorder, and disorganized (or) abnormal behaviour) and neuropsychiatric symptoms (sleep disturbances, hostility (or) aggression, confusion, affective symptoms, anxiety symptoms, and cognitive difficulties) were the frequently observed symptoms along with delusion and hallucination in the included cases. These findings are consistent with earlier literature [9, 66], which identified comparable symptom patterns in patients with drug-induced psychosis, including drugs such as aminoglycosides, other antitubercular agents, beta-lactam agents, and psychotropic drugs (central nervous system (CNS) stimulants, and CNS depressants) [9, 66]. However, they might serve as significant early-stage indicators and call for further research.

A high frequency of patients had a psychomotor disturbance in our study. Pyridoxine deficiency has been reported to enhance noradrenergic signaling and facilitate behavioural deficits in mice [67]. Pyridoxine (100 mg/day) improved neuroleptic-induced parkinsonism and psychotic symptoms in a patient with schizophrenia [68]. A double-blind placebo-controlled cross-over study in patients with schizophrenia reported pyridoxine as an effective agent for tardive dyskinesia at doses from 300 mg/day [69]. A randomized, double-blind, placebo-controlled study reported that the high-dose pyridoxine-treated patients (1200 mg/day administered as 600 mg BID) had improvement in neuroleptic-induced akathisia symptoms within a few days [70]. The quality of evidence about the effectiveness of pyridoxine for the treatment of neuroleptic-induced tardive dyskinesia among people with schizophrenia and other related psychotic disorders is low [71]. Another study reported that though pyridoxine may be beneficial for ameliorating antipsychotic-induced akathisia, there was no significant difference observed among those who received a low dose (600 mg/day administered as 300 mg BID) or high dose (1200 mg/day administered as 600 mg BID) pyridoxine [72]. Pyridoxine 100 mg was the most prescribed dose; however, several patients have been prescribed pyridoxine doses lesser than 100 mg/day post-isoniazid-induced psychosis.

According to Chan et al., even though pyridoxine supplementation is recommended in patients who are at risk of neuropathy due to underlying medical conditions such as diabetes, uraemia, alcoholism, and HIV infection, it is not an effective treatment for isoniazid-induced psychosis [73]. On the other hand, Yadav et al. suggest that pyridoxine supplementation should be considered after the occurrence of isoniazid-induced psychosis to ensure the reintroduction of isoniazid does not exacerbate psychosis [74]. Only a single study had administered a high dose of pyridoxine of 1000 mg/day (administered as 500 mg intramuscularly BID for 4 days), which, however, did not improve psychosis during the period of administration [25]. The role of pyridoxine supplementation for isoniazid-induced psychosis warrants robust scientific validation for conclusive recommendations. A comparative benefit assessment of different doses of pyridoxine supplementation for the treatment of isoniazid-induced psychosis is also required. Our systematic review shows that approximately 60% (*n* = 23) of patients have received psychotropic drugs. Notably, in our review, a substantial proportion of the cases did not involve the administration of psychotropic drugs to treat isoniazid-induced psychosis. Among the psychotropics used, lorazepam, which belongs to the class of benzodiazepines [75], is the most frequently administered. The other antipsychotics, which belong to the classes butyrophenones and atypical antipsychotics [76], such as haloperidol, risperidone, quetiapine, and olanzapine, were employed in varying frequencies to treat the psychotic symptoms. In our review, isoniazid-induced psychosis was assessed using numerous ADR assessment tools across the studies, with the WHO UMC scale and the Naranjo scale being the most frequently used. Using different causality assessment tools underscores the importance of standard approaches to establishing the likelihood of isoniazid-induced psychosis [77].

Our systematic review has a few inherent limitations that should be considered. Our review is primarily limited to case reports and case series, which may not provide the strongest degree of evidence in contrast to other study designs. Additionally, by only including articles in English, it is possible that pertinent studies published in different languages were left out. Due to the smaller number of reports and several missing data in these reports, our analysis could affect the precision and generalizability of the findings. Participants with a current or previous history of psychiatric illness studies were excluded from the study. We also excluded grey literature in our review. Some relevant case reports could not be retrieved because the full text was unavailable in the databases. Some case reports have scanty descriptions of the symptoms. However, we included these articles as they mentioned the evaluation of the patient by a mental health professional that led to the diagnosis of isoniazid-induced psychosis. Ascertaining the psychosis diagnosis becomes even more challenging, especially in studies involving the paediatric population. The classificatory systems in psychiatry do get revised over time. The importance of certain symptoms would change with the changing diagnostic guidelines. Including case reports from earlier decades, such as the 1950s, and applying the current classificatory guidelines to relook at the clinical manifestations would have some limitations. We restricted our attention to the relationship between isoniazid and psychosis, which potentially led to an incomplete understanding of the broader mental health implications, such as anxiety and depression. These limitations raise attention to the need for further research to address these gaps and offer a more thorough understanding of the effects of isoniazid on mental health. Future research should focus on well-designed epidemiological studies to determine the incidence/prevalence of isoniazid-induced psychosis and neuropsychiatric symptoms. Our review provides evidence for closer monitoring of psychotic symptoms during the first 2 months of isoniazid treatment as shown in Fig. 3, with a rechallenge of isoniazid resulting in the recurrence of the psychotic and/or neuropsychiatric symptoms in the majority of the patients. However, we could not conclusively conclude on the risk factors that may lead to isoniazid induced psychosis as well as on the clinical utility and dosage recommendations of pyridoxine as a therapeutic agent for the management of isoniazid-induced psychosis symptoms. Further research may focus on retrieval of a greater number of isoniazid-induced psychosis and neuropsychiatric symptoms cases using pharmacovigilance databases such as VigiBase (WHO global database of individual case safety reports), EudraVigilance (European pharmacovigilance database), and the Food and Drug (FDA) adverse event reporting system (FAERS), which may also include unpublished cases for in-depth analysis of the risk factors, severity assessment, and signal detection [78].

## Conclusion

Our systematic review identified a higher frequency of isoniazid-induced psychosis during the first 2 months of TB treatment, with a relatively early onset observed among patients of 18 years or less, warranting the need for closer monitoring during this period. Individual patient demographic, social, and clinical characteristics may influence the manifestation of this adverse effect. There is a need for well-controlled studies, to evaluate the influence of genetic variants and isoniazid pharmacokinetics with the development of isoniazid-induced psychosis symptoms and for optimizing pyridoxine therapy.

## Supplementary Information

Below is the link to the electronic supplementary material.

Supplemental Table 1 (DOCX 29 kb)

Supplemental Table 2 (DOCX 14 kb)

Supplemental Fig. 1 Isoniazid induced psychosis symptoms profile. Footnote: * Cases that were not isoniazid induced psychosis has been excluded from the case series. Abbreviation: Form_tht_dis: Formal thought disorder (PNG 153 kb)

High resolution image (TIF 692 kb)

Supplemental Fig. 2 Quality assessment of included studies. Footnote: ^1^Tool for evaluating the methodological quality of case reports and case series developed by Murad et al. ^2^* Cases that were not isoniazid induced psychosis has been excluded from the case series. ^3^ Q1: Does the patient(s) represent(s) the whole experience of the investigator (centre) or is the selection method unclear to the extent that other patients with similar presentation may not have been reported? Q2: Was the exposure adequately ascertained? Q3: Was the outcome adequately ascertained? Q4: Were other alternative causes that may explain the observation ruled out? Q5: Was there a challenge/rechallenge phenomenon? Q6: Was there a dose-response effect? Q7: Is the case(s) described with sufficient details to allow other investigators to replicate the research or to allow practitioners make inferences related to their own practice? Abbreviation: Q: Question (PNG 134 kb)

High resolution image (TIF 528 kb)

## Data Availability

No datasets were generated or analyzed during the current study.
