# Supplementary material for: Characteristics of isoniazid-induced psychosis: a systematic review of case reports and case series
Source: Eur J Clin Pharmacol. 2024 Aug 13;80(11):1725–40. doi: 10.1007/s00228-024-03738-x (PMC11458663; doi:10.1007/s00228-024-03738-x)
Supplement: Supplementary file 2 — (DOCX 14 kb) [file 228_2024_3738_MOESM2_ESM.docx]

**Supplemental Table 2. Search strategy**

Search done: From the date of inception of the database to June 2024

| **S.N** | **Databases** | **Query** | **Results** |
| --- | --- | --- | --- |
| 1 | Embase | ('isoniazid’ OR ‘inh’ OR 'anti-tb drugs' OR 'anti-tb agents') AND ('psychosis' OR 'psychoses' OR 'schizophrenia spectrum and other psychotic disorders' OR ‘Schizophrenia’ OR 'schizoid disorder' OR 'schizoaffective disorder' OR ‘delusion*’ OR ‘hallucination*’ OR 'mania*' OR 'depression*' OR 'bipolar disorder*' OR 'bipolar related disorders*' OR 'behavioral disorders*' OR 'delirium' OR 'encephalopathy') | 256 |
| 2 | PubMed | ('isoniazid’ OR ‘inh’ OR 'anti-tb drugs' OR 'anti-tb agents') AND ('psychosis' OR 'psychoses' OR 'schizophrenia spectrum and other psychotic disorders' OR ‘Schizophrenia’ OR 'schizoid disorder' OR 'schizoaffective disorder' OR ‘delusion*’ OR ‘hallucination*’ OR 'mania*' OR 'depression*' OR 'bipolar disorder*' OR 'bipolar related disorders*' OR 'behavioral disorders*' OR 'delirium' OR 'encephalopathy') | 44 |
| 3 | Scopus | ('isoniazid’ OR ‘inh’ OR 'anti-tb drugs' OR 'anti-tb agents') AND ('psychosis' OR 'psychoses' OR 'schizophrenia spectrum and other psychotic disorders' OR ‘Schizophrenia’ OR 'schizoid disorder' OR 'schizoaffective disorder' OR ‘delusion*’ OR ‘hallucination*’ OR 'mania*' OR 'depression*' OR 'bipolar disorder*' OR 'bipolar related disorders*' OR 'behavioral disorders*' OR 'delirium' OR 'encephalopathy') | 422 |
