# Supplementary figures and images for: Characteristics of isoniazid-induced psychosis: a systematic review of case reports and case series
Source: Eur J Clin Pharmacol. 2024 Aug 13;80(11):1725–40. doi: 10.1007/s00228-024-03738-x (PMC11458663; doi:10.1007/s00228-024-03738-x)

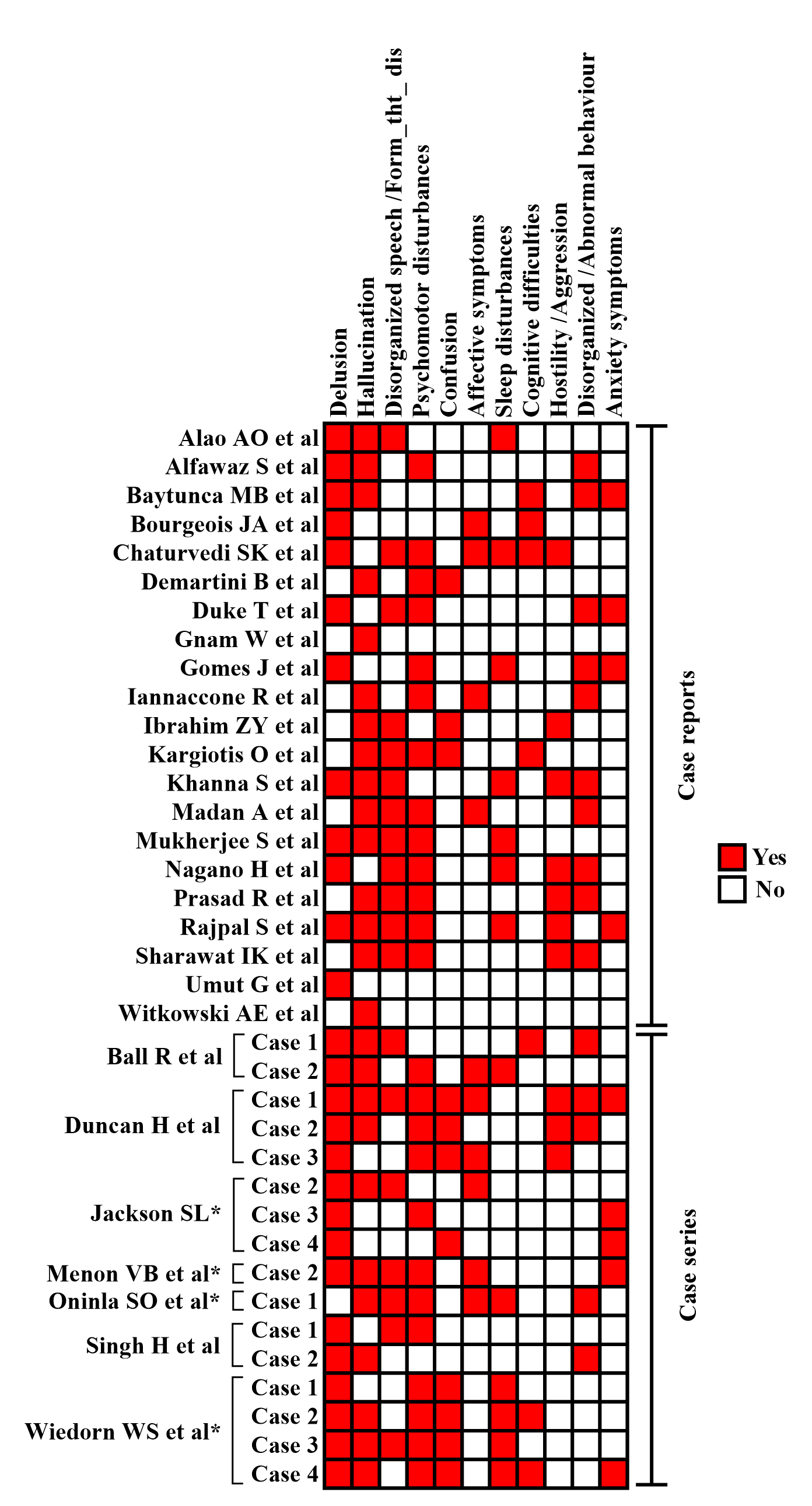

Supplement: Supplementary file 3 — Isoniazid induced psychosis symptoms profile. Footnote: * Cases that were not isoniazid induced psychosis has been excluded from the case series. Abbreviation: Form_tht_dis: Formal thought disorder (PNG 153 kb) [file 228_2024_3738_Fig4_ESM.png]

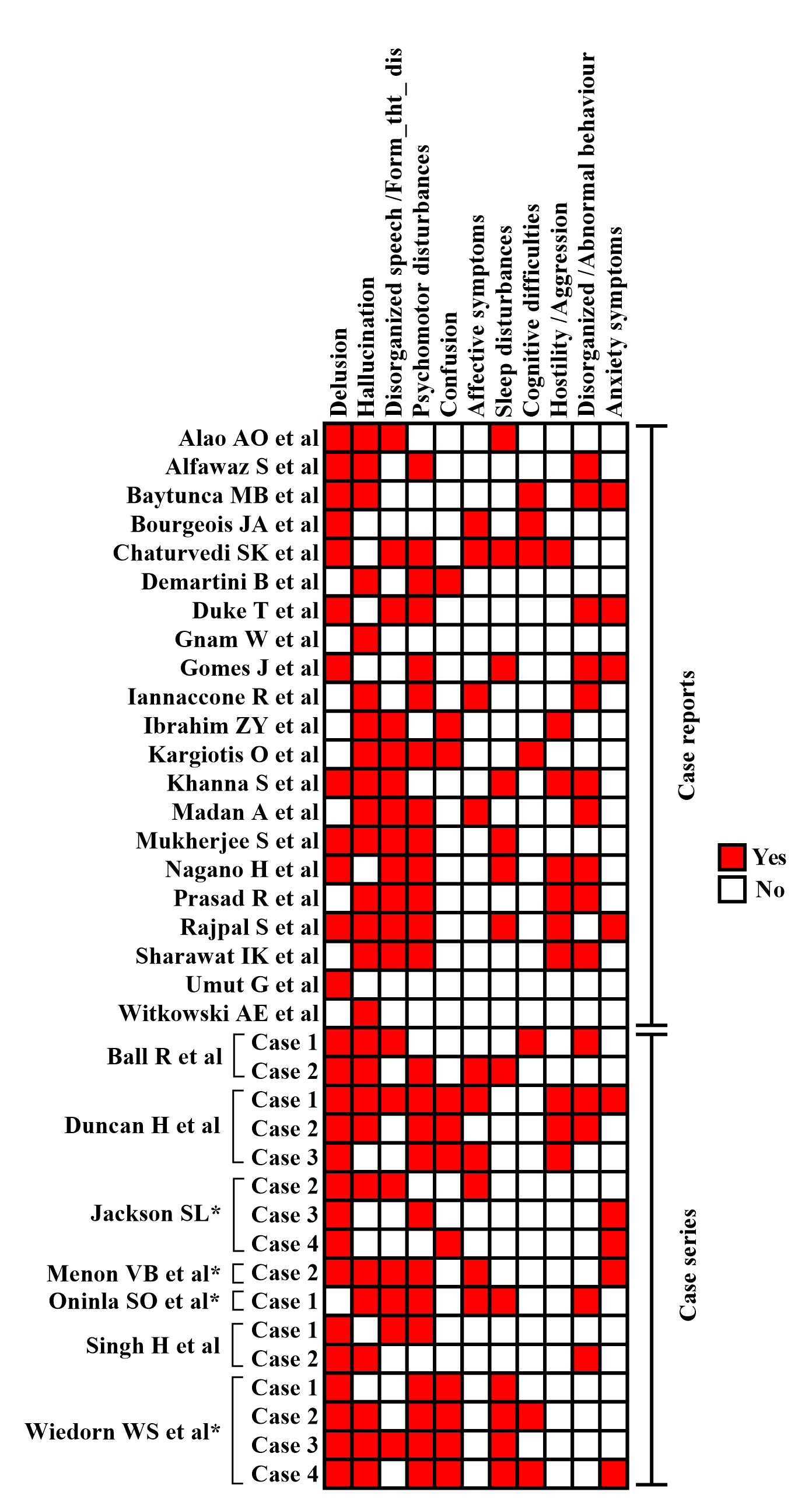

Supplement: Supplementary file 4 — High resolution image (TIF 692 kb) [file 228_2024_3738_MOESM3_ESM.tif]

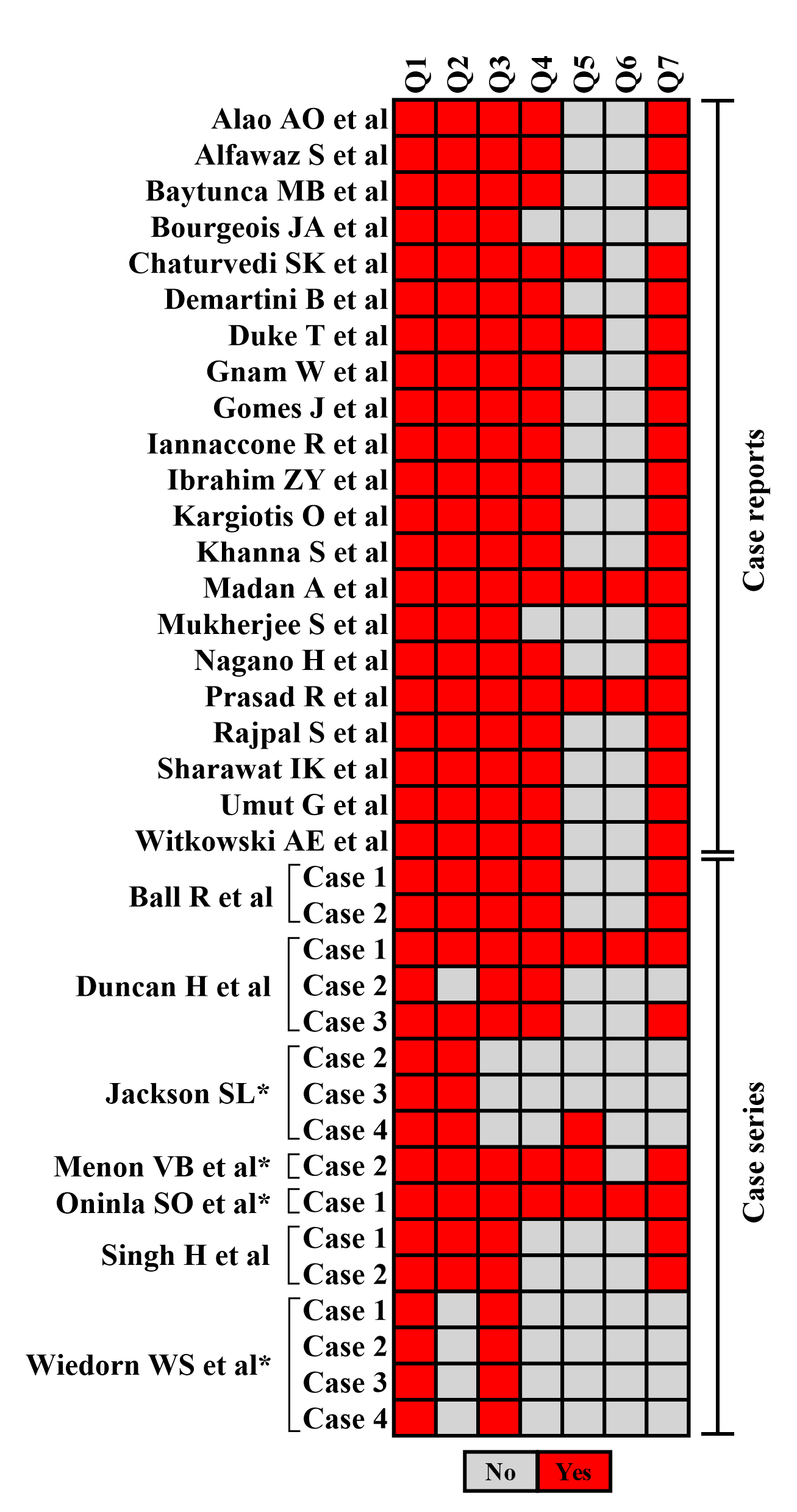

Supplement: Supplementary file 5 — Quality assessment of included studies. Footnote: 1Tool for evaluating the methodological quality of case reports and case series developed by Murad et al. 2* Cases that were not isoniazid induced psychosis has been excluded from the case series. 3 Q1: Does the patient(s) represent(s) the whole experience of the investigator (centre) or is the selection method unclear to the extent that other patients with similar presentation may not have been reported? Q2: Was the exposure adequately ascertained? Q3: Was the outcome adequately ascertained? Q4: Were other alternative causes that may explain the observation ruled out? Q5: Was there a challenge/rechallenge phenomenon? Q6: Was there a dose-response effect? Q7: Is the case(s) described with sufficient details to allow other investigators to replicate the research or to allow practitioners make inferences related to their own practice? Abbreviation: Q: Question (PNG 134 kb) [file 228_2024_3738_Fig5_ESM.png]

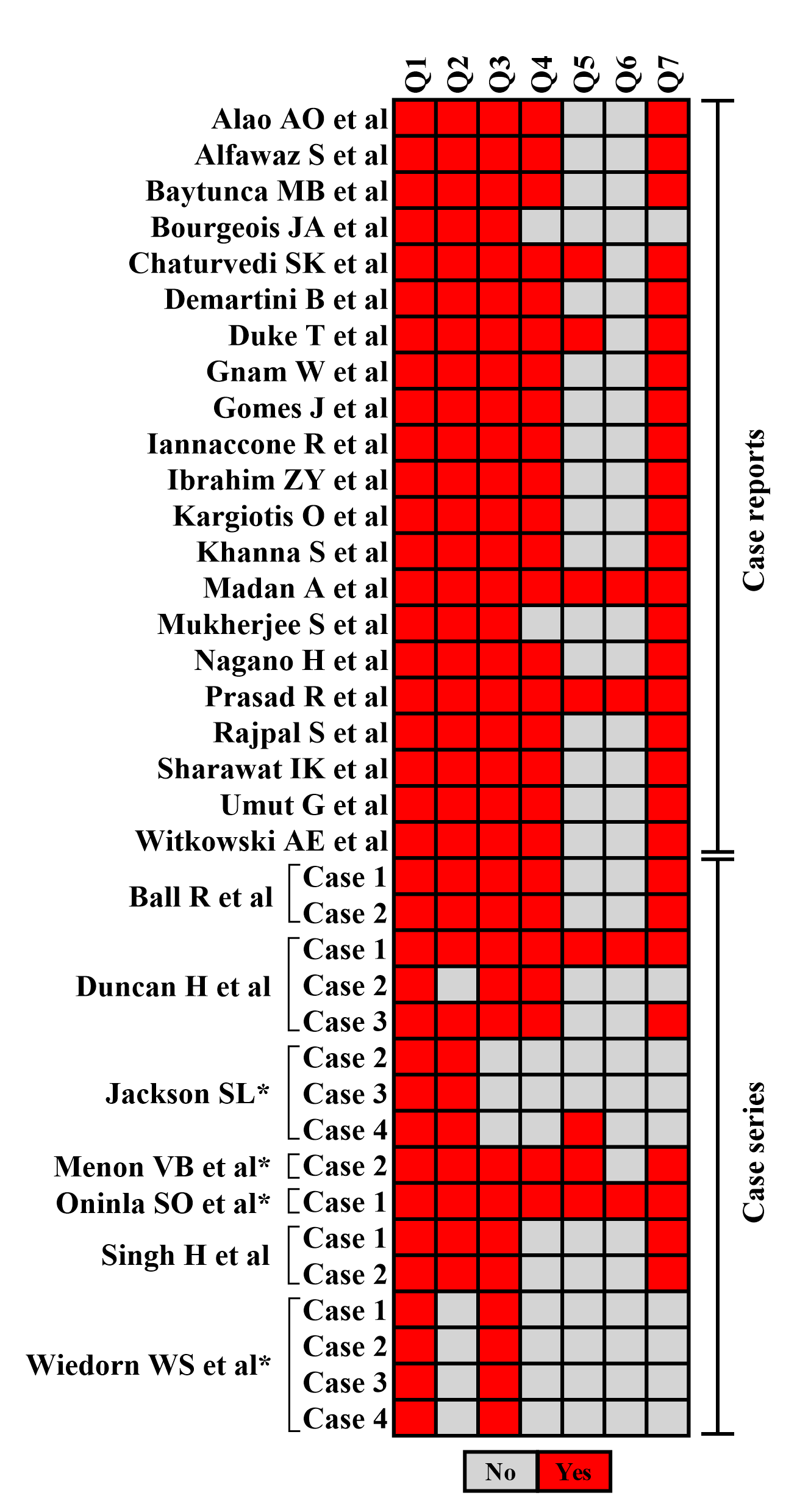

Supplement: Supplementary file 6 — High resolution image (TIF 528 kb) [file 228_2024_3738_MOESM4_ESM.tif]
